# A Simple, Quick and Eco-Friendly Strategy of Synthesis Nanosized α-LiFeO_2_ Cathode with Excellent Electrochemical Performance for Lithium-Ion Batteries

**DOI:** 10.3390/ma11071176

**Published:** 2018-07-10

**Authors:** Youzuo Hu, Hongyuan Zhao, Xingquan Liu

**Affiliations:** R&D Center for New Energy Materials and Integrated Energy Devices, State Key Laboratory of Electronic Thin Film and Integrated Devices, University of Electronic Science and Technology of China, Chengdu 610054, China; huyouzuo@126.com

**Keywords:** α-LiFeO_2_, nanoparticle, cathode material, ambient temperature

## Abstract

Nanosized α-LiFeO_2_ samples were successfully synthesized via a simple, quick and eco-friendly strategy at ambient temperature followed by a low temperature calcined process. X-ray diffraction (XRD), scanning electronic microscopy (SEM) and transmission electron microscopy (TEM) measurements revealed that the optimal α-LiFeO_2_ sample was composed of extremely small nanoparticles. The electrochemical properties were tested at 0.1 C in the cut-off voltage of 1.5–4.8 V. The sample obtained at 150 °C for 6 h exhibited the best cycling stability with high initial discharge capacity of 223.2 mAh/g, which was extremely high for pristine α-LiFeO_2_ without any modification process. After 50 cycles, the discharge capacity could still maintain 194.5 mAh/g with good capacity retention. When the charge–discharge rate increased to 0.2 C and 0.5 C, the initial discharge capacities were 216.6 mAh/g and 171.5 mAh/g, respectively. Furthermore, the optimal sample showed low charge transfer resistance and high lithium-ion diffusion coefficients, which facilitated the excellent electrochemical performance.

## 1. Introduction

Lithium-ion batteries have been widely applied to laptop computers, electric vehicles, hybrid vehicles, and other portable electronic products [1]. As an important part of lithium-ion batteries, cathode materials largely determine the electrochemical performance [2]. Among all the alternate cathode materials, LiFeO_2_ with layered rock-salt structure has attracted significant attention for its high theoretical capacity of 282 mAh/g, abundant raw materials, low cost, and nontoxic [2,3]. According to the reported results, LiFeO_2_ has many structural forms, such as α-, β- and γ-conjugated forms [4,5]. Among them, α-LiFeO_2_ has attracted more attention due to the simpler synthesis method and excellent electrochemical properties [6]. Many researchers have paid great efforts to synthesized α-LiFeO_2_ via different methods. Sakurai et al. synthesized α-LiFeO_2_ sample by high temperature solid-state method, but the sample was proven to be electrochemically inactive [7]. Then, they successfully synthesized active α-LiFeO_2_ sample at low temperature of less than 250 °C via H^+^/Li^+^ ionic exchange reaction. Although this sample was electrochemically active, it exhibited only reversible capacity of 80 mAh/g and poor cycle performance at 0.1 C [8]. Furthermore, α-LiFeO_2_ synthesized by Li et al. via solvothermal methods exhibited higher initial capacity of 135 mAh/g, but the cycling performance was still poor [9]. To further enhance the cycling performance, Rahman et al. prepared α-LiFeO_2_-MWCNT compounds via radio frequency oxygen plasma method [10]. The optimal compound showed higher initial capacity of 236 mAh/g and better cycling performance, but the synthetic route is too complicated and costly to commercialize. Therefore, it is necessary to develop a more appropriate way to prepare α-LiFeO_2_ with excellent electrochemical performance.

According to the existing literature [11], nanomaterials can make a significant impact on the electrochemical performance of cathodes as the reduced particle size contributes to the full contact of cathode material and electrolyte and shortens Li^+^ transportation distance. For α-LiFeO_2_, the electrochemical performance is significantly impaired by the poor conductivity. Thus, reducing the particle size is a valid approach to promote the ionic transportation and the electronic conductivity. Morales et al. [12] synthesized α-LiFeO_2_ nanoparticles with smaller average diameter of about 50 nm. This material could exhibit high initial specific capacity of 150 mAh/g. Hirayama et al. further confirmed the improvement effect of smaller particle size on the electrochemical performance of LiFeO_2_ [13]. However, it is important to note that the improved performance of α-LiFeO_2_ nanoparticles reported in the above literature still cannot meet the demands of high reversible capacity of cathode materials. To further enhance the reversible capacity, Wang et al. synthesized α-LiFeO_2_ nanoparticles with particle size under 10 nm. It showed extremely high initial specific capacity of 290.6 mAh/g at 0.1 C, but the cycling performance was not very satisfactory for cathode materials [14]. Based on the above analysis, it can be speculated that the reduction of particle size may present excellent electrochemical performance.

We successfully synthesized nanosized α-LiFeO_2_ samples via a simple, quick and eco-friendly strategy at ambient temperature followed by a low temperature calcine process. Compared with the reported methods [8,15,16,17,18,19], the present synthesis strategy not only reduces the production cost of α-LiFeO_2_ with high performance, but also alleviates the environment pollution. More importantly, the optimal α-LiFeO_2_ sample with small particle size showed excellent electrochemical properties at high voltage.

## 2. Experimental

### 2.1. Synthesis of α-LiFeO_2_ Cathode Materials

The α-LiFeO_2_ nanoparticles were prepared by a simple, quick and eco-friendly strategy followed by a low temperature calcined process. Firstly, 2.73 g LiOH·H_2_O and 3 g Fe(NO)_3_·9H_2_O was dissolved in 20 mL ethanol. The suspension was intensely stirred under magnetic stirring at ambient temperature for 5 h. After being washed with distilled water and ethanol repeatedly to eliminate redundant lithium hydrate, the brown product was dried at 80 °C for 12 h in an air oven. The brown particles were obtained by hand-grinding in an agate mortar. Finally, the product was sintered at different temperatures (100 °C, 150 °C, 250 °C, and 350 °C) for 6 h in tube furnace under argon atmosphere.

### 2.2. Characterizations of α-LiFeO_2_ Cathode Materials

The crystalline phase was characterized by X-ray diffraction (XRD, Bruker DX-1000, Bruker, Karlsruhe, Germany, Cu Kα radiation). The particle morphology of the α-LiFeO_2_ powders was examined with scanning electron microscope (SEM, JEOL, JSM-6360LV, Tokyo, Japan) and transmission electron microscopy (TEM, JEOL, JEM-3010, Tokyo, Japan). The specific surface area and pore volume were obtained by Brunauer–Emmett–Teller (BET) N_2_ adsorption–desorption measurement (BET, NOVA3000 analyzer, Quantachrome Instrument, Boynton Beach, FL, USA). X-ray photoelectron spectroscopy (XPS, ESCALAB 250XI, Thermo Fisher Scientific, Boston, MA, USA) was used to estimate the Valence states of α-LiFeO_2_ electrodes during different process.

### 2.3. Electrochemical Tests of α-LiFeO_2_ Cathode Materials

The electrochemical performance of α-LiFeO_2_ were evaluated at ambient temperature. The positive electrode was prepared with 85% synthesized product, 10% acetylene black, and 5% polyvinylidene fluoride binder in *N*-methyl-2-pyrrolidone solvent. The slurry was pressed onto Al foil and dried at 100 °C for 4 h and then punched into round positive slices with 12 mm diameter. The lithium foil was the anode electrode, ehilr celgard microporous polypropylene membrane was the diaphragm. The electrolyte solution was 1 M lithium hexafluorophosphate (LiPF6) solution in a mixture of the ethylene carbonate (EC) and diethl carbonate (DEC) in a 1:1 volume ratio. The charge–discharge profiles were investigated by Land CT2001A battery testing system with a voltage window of 1.5–4.8 V. The EIS was studied by CS-350 electrochemical workstation with frequency ranging from 0.1 Hz to 100 kHz. The CV was also tested by CS-350 electrochemical workstation, with scan rate of 0.1 mV/s.

## 3. Results and Discussion

**Scheme 1:**
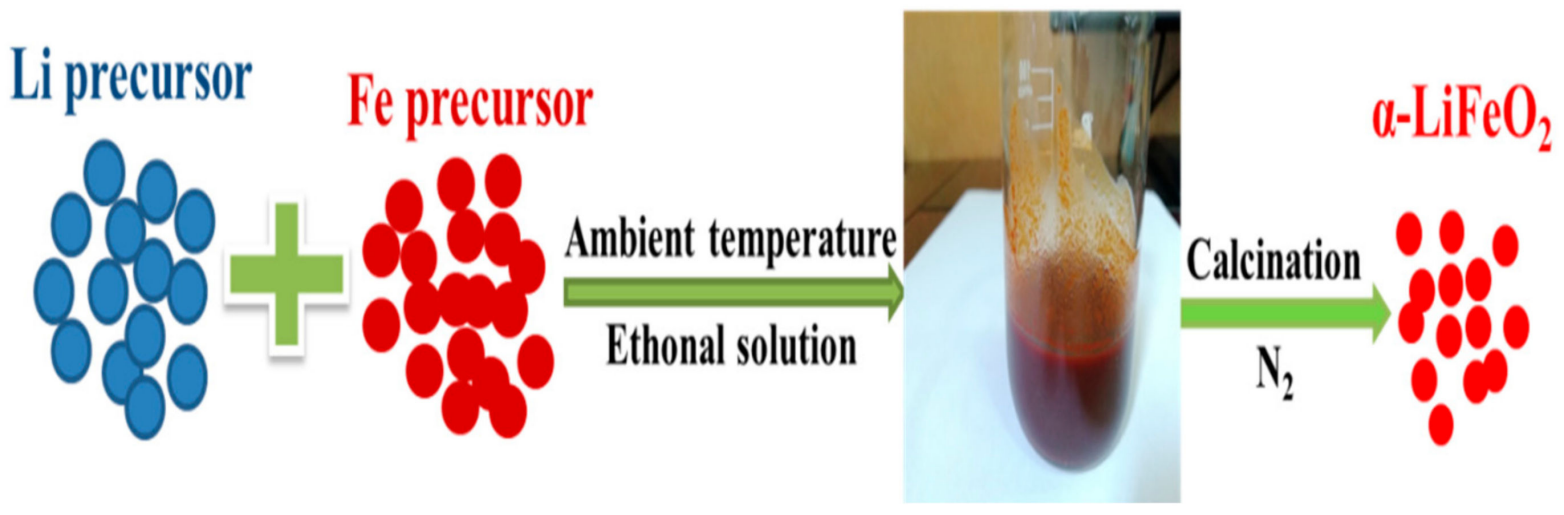
Schematic illustration of the synthesis process for composite α-LiFeO_2_ nanoparticles.

Scheme 1 illustrates the synthesis process of α-LiFeO_2_, where the Li precursor and Fe precursor dissolved in ethanol solution and reacted together under ambient temperature. Herein, the formation of α-LiFeO_2_ includes two stages, the formation of nucleuses and the growth of α-LiFeO_2_ crystals. Meanwhile, the low temperature calcination process further improved the crystallinity of α-LiFeO_2_.

**Figure 1:**
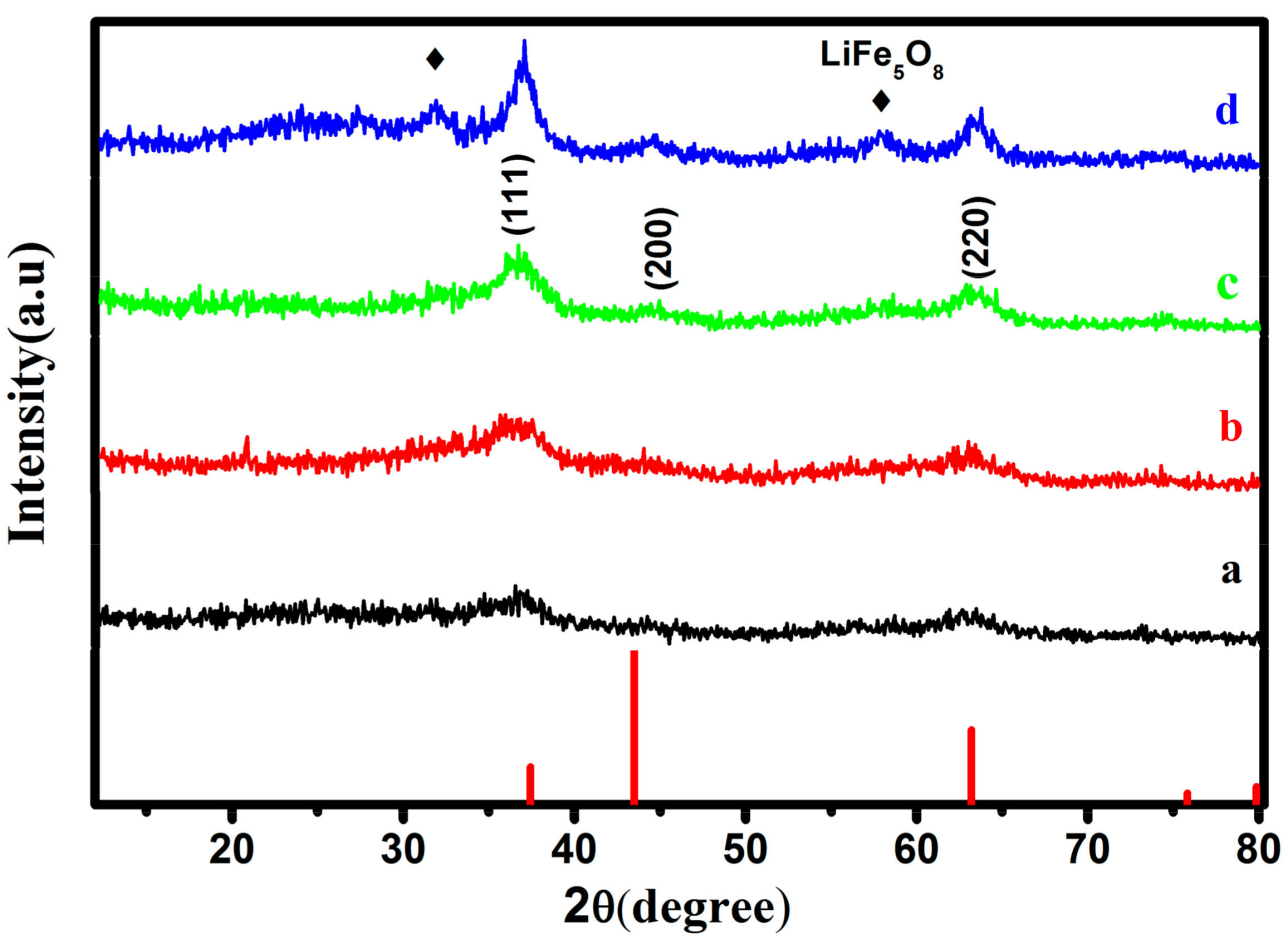
XRD patterns of the α-LiFeO_2_ samples obtained at various temperatures: (**a**) 100 °C; (**b**) 150 °C; (**c**) 250 °C; and (**d**) 350 °C.

The XRD patterns of the α-LiFeO_2_ samples obtained under various temperatures for 6 h in argon atmosphere is presented in Figure 1. When the temperature is below 250 °C, the diffraction peaks of the obtained samples match the standard reflection peaks of α-LiFeO_2_ well (Space group Fm3m, JCPDS No. 74-2283, a = 4.156 Å). When the temperature rises to 350 °C, there is an impurity phase, which can be attributed to spinel LiFe_5_O_8_ [20,21]. This result agrees with the reported result that the LiFe_5_O_8_ impure phase appears after high temperature sinter process [15]. However, with luck, the α-LiFeO_2_ would still be the main phase, and the (110), (200) and (220) diffraction peaks gradually become thinner upon increasing temperature.

**Figure 2:**
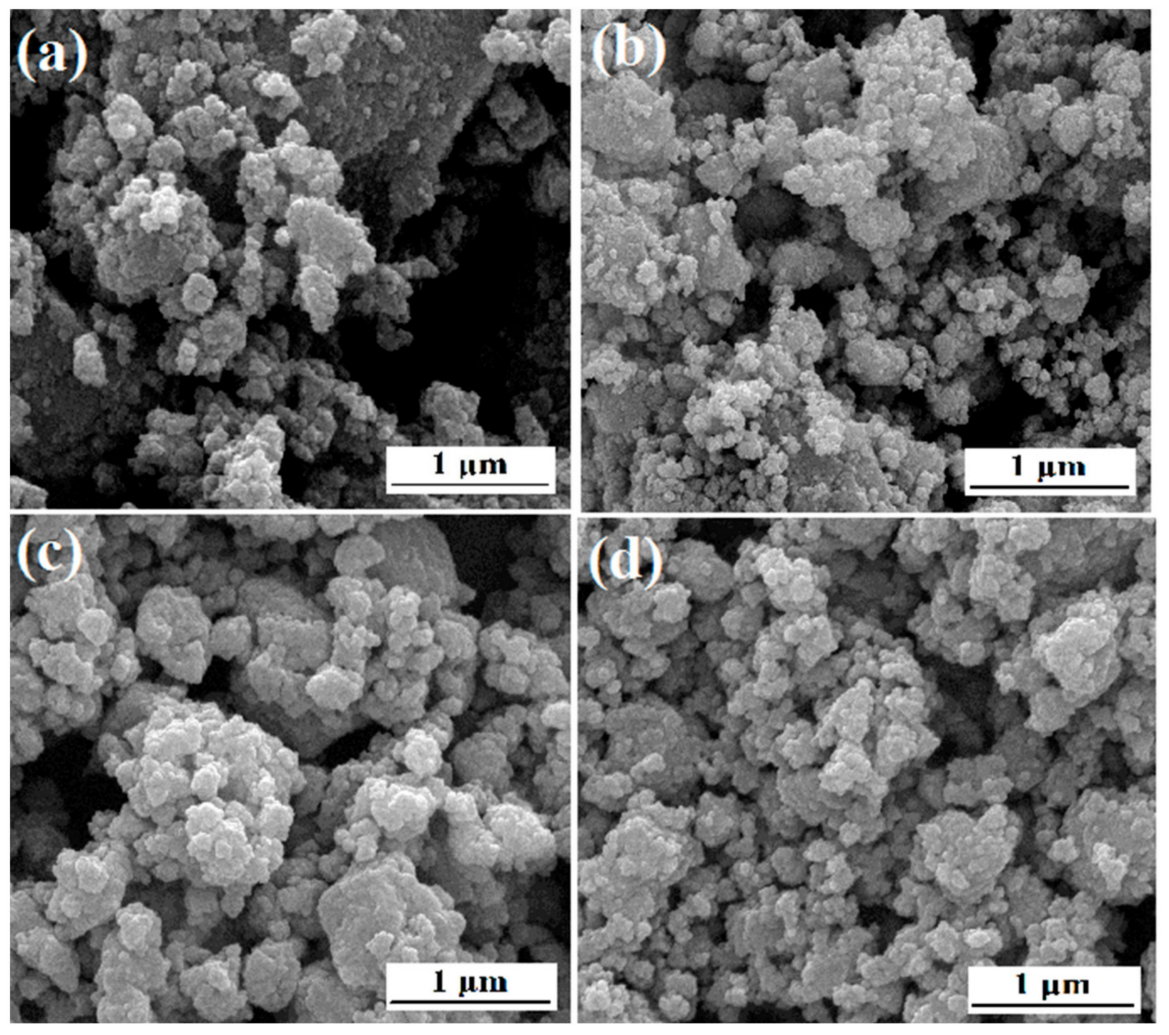
SEM images of α-LiFeO_2_ nanoparticles synthesized at different temperatures: (**a**) 100 °C; (**b**) 150 °C; (**c**) 250 °C; and (**d**) 350 °C.

**Figure 3:**
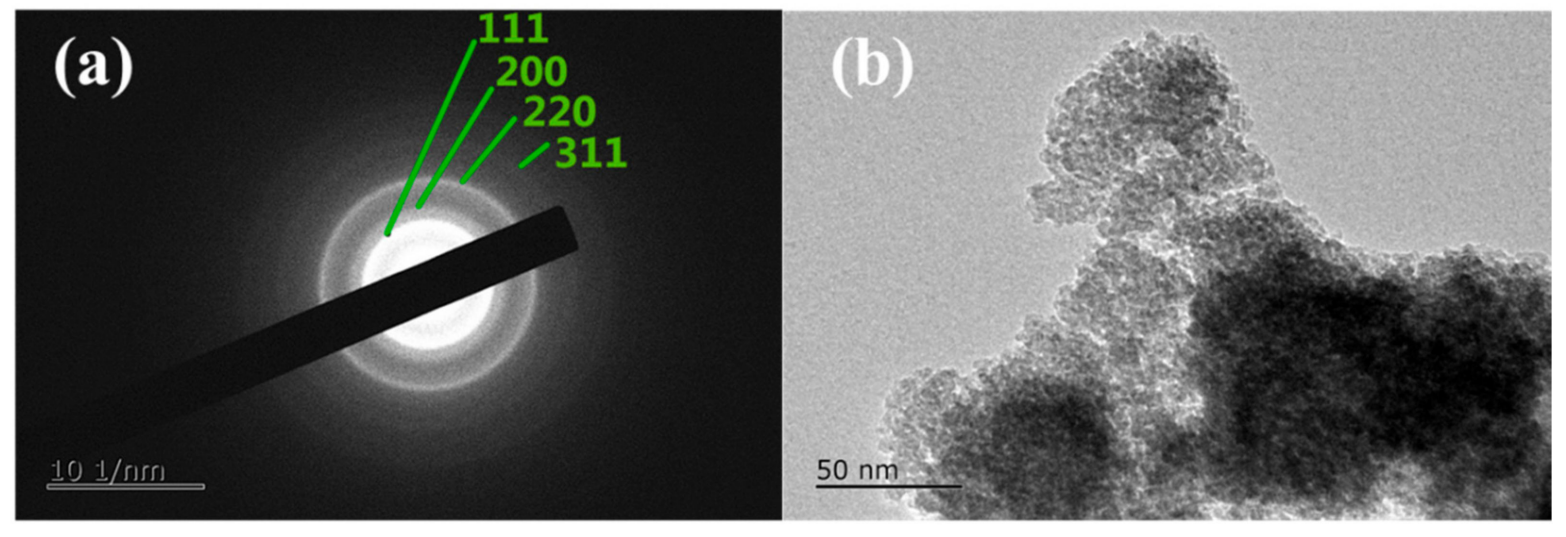

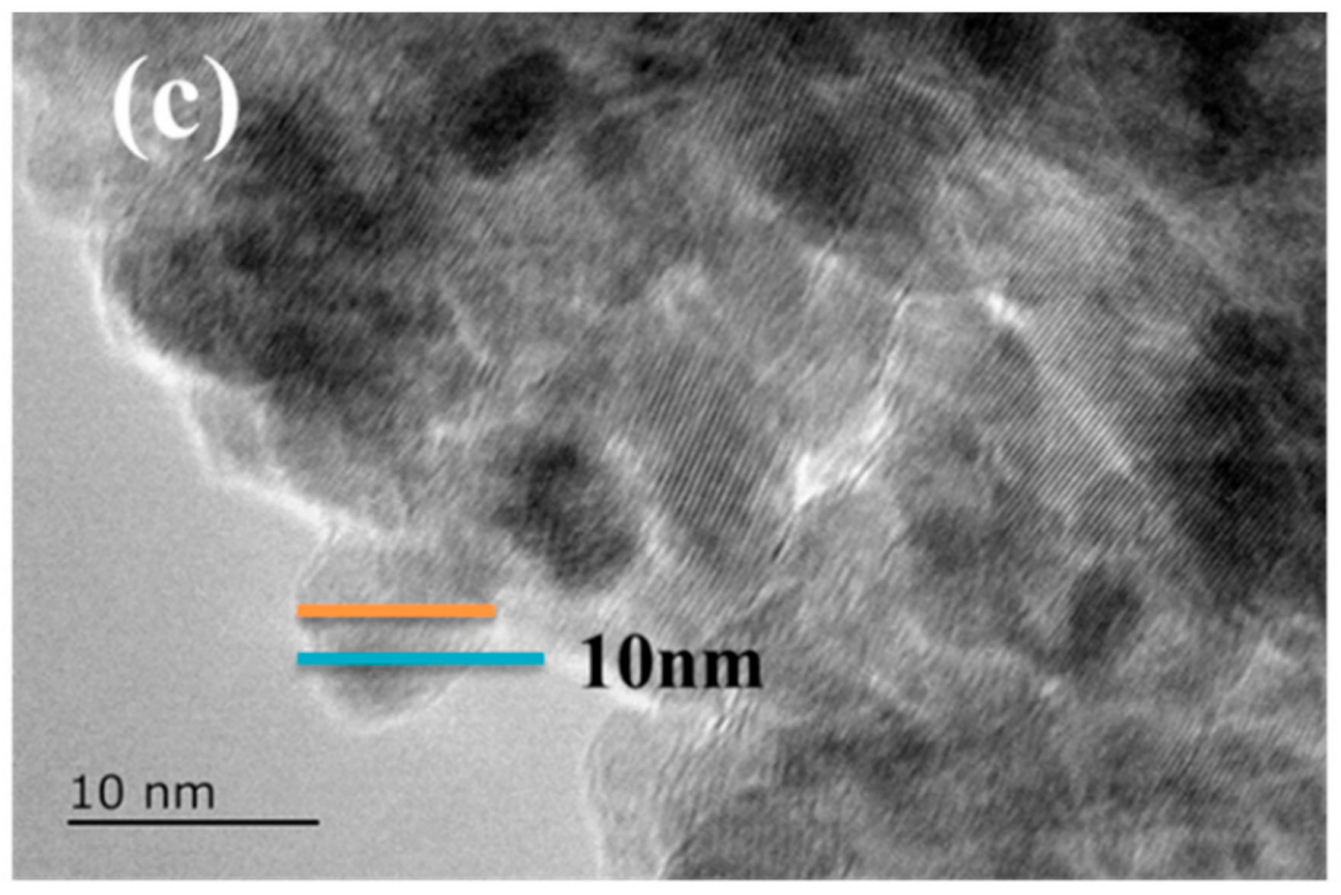
(**a**) Selected area electron diffraction pattern; and (**b**,**c**) TEM image and of the sample obtained at 150 °C.

Figure 2 presents the SEM measurements of the α-LiFeO_2_ nanoparticles obtained at various temperatures. As shown in Figure 2, the sintering temperature does not have a significant impact on the morphology and particle distribution. For these samples, the particle size and grain distribution are roughly the same. Each sample is composed with round likely ultra-small particles. Figure 3a–c shows the SAED image and TEM images of the sample obtained at 150 °C. As shown in Figure 3a, the diffraction rings of (200), (220), (222), and (311) can be indexed to cubic α-LiFeO_2_ [22]. Furthermore, Figure 3b,c shows the sample with even particle size distribution and the average particle size is below 10 nm. We can conclude from the SEM and TEM images that α-LiFeO_2_ particles tended to agglomerate a bunch-like morphology. Morales et al. synthesized 50 nm α-LiFeO_2_ nanoparticles via molten-salt way that also agglomerated with a bunch-like morphology, and they believed such particular architecture can provide an uninterrupted pathway for lithium ion diffusion, which enhances the electrochemical properties [10].

**Figure 4:**
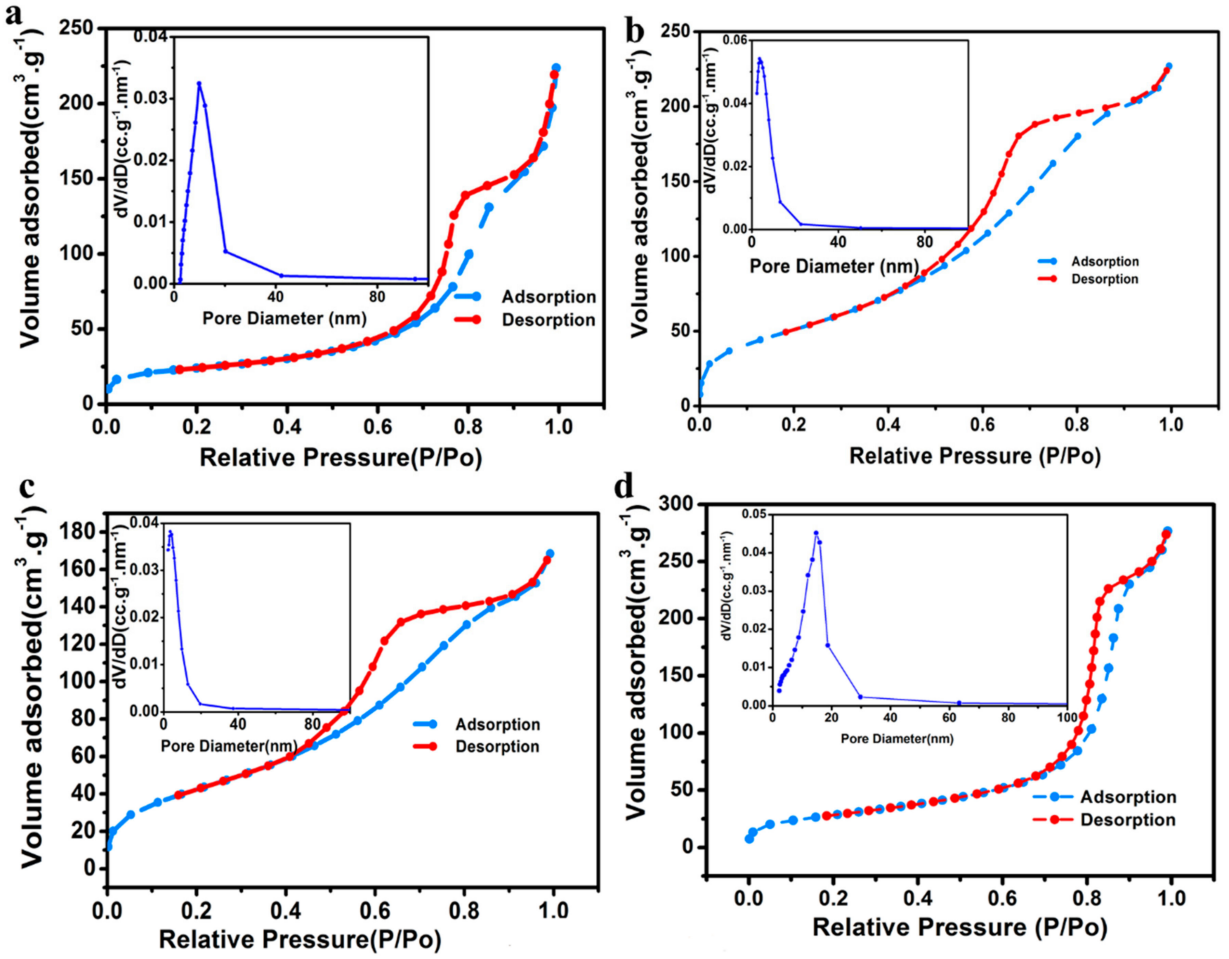
N_2_ adsorption–desorption isotherms (BET) and pore-size (BJH) diagram for α-LiFeO_2_ nanoparticles synthesized at different temperatures: (**a**) 100 °C; (**b**) 150 °C; (**c**) 250 °C; and (**d**) 350 °C.

The N_2_ adsorption–desorption measurements were introduced to obtain the surface information of α-LiFeO_2_ calcined at different temperatures. In Figure 4 and Table 1, the specific surface areas of samples calcined at 100 °C, 150 °C, 250 °C and 350 °C are 82.11 m^2^·g^−1^, 194.48 m^2^·g^−1^, 159.30 m^2^·g^−1^ and 103.42 m^2^·g^−1^, respectively. Furthermore, the corresponding desorption average pore diameter of these samples are 8.46 nm, 3.27 nm, 3.61 nm and 8.28 nm, respectively. Clearly, α-LiFeO_2_ calcined at 150 °C possesses the largest specific surface area and smallest pore diameter; these surface structures supply a large specific surface area to enhance the contact area between electrolyte and electrode, and shorten the transportation distance of Li^+^ ions between the nanoparticles [23].

**Figure 5:**
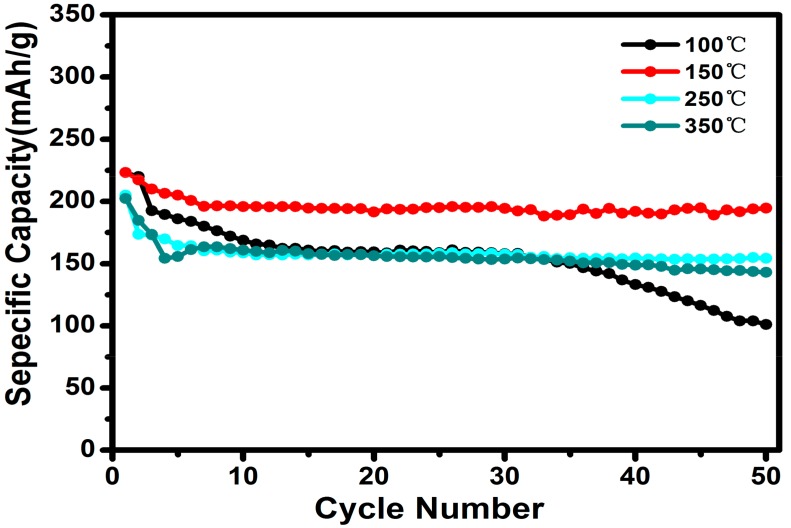
Cycling performance of the resulting LiFeO_2_ nanoparticles obtained at different temperature.

The cycling performance of the electrodes obtained at different temperature are shown in Figure 5. The first specific capacities of these electrodes are 223.2 mAh/g, 223.3 mAh/g, 205 mAh/g, and 202.3 mAh/g with capacity retention of 45.2%, 87.1%, 75.3%, and 70%, respectively. The cycling performance is much better than that of the reported pure phase α-LiFeO_2_ [16,23,24]. The capacity decreased as the temperatures increase, which might be attributed to the higher sintering temperature leading to the impurity phase of LiFe_5_O_8_, which is considered responsible for the sharp decrease of initial discharge capacity [25,26]. Furthermore, Sakurai found that α-LiFeO_2_ synthesized at high temperature was electrochemically inactive, as the higher temperature may increase the disorder degree of iron ions on lithium sites and block lithium diffusion pathways [8,27,28].

Among these samples, the sample obtained at 150 °C exhibits the highest initial specific capacity of 223.3 mAh/g. More importantly, the discharge capacity retains 194.5 mAh/g after 50 cycles. Although there is a sharp capacity decline in the first five cycles, this sample can show outstanding cycling performance with high capacity retention of 96.9% in subsequent charge–discharge processes. The sample calcined at 150 °C exhibited such excellent electrochemical performance may attributed to small average particle size of below 10 nm and lower calcined temperature.

**Figure 6:**
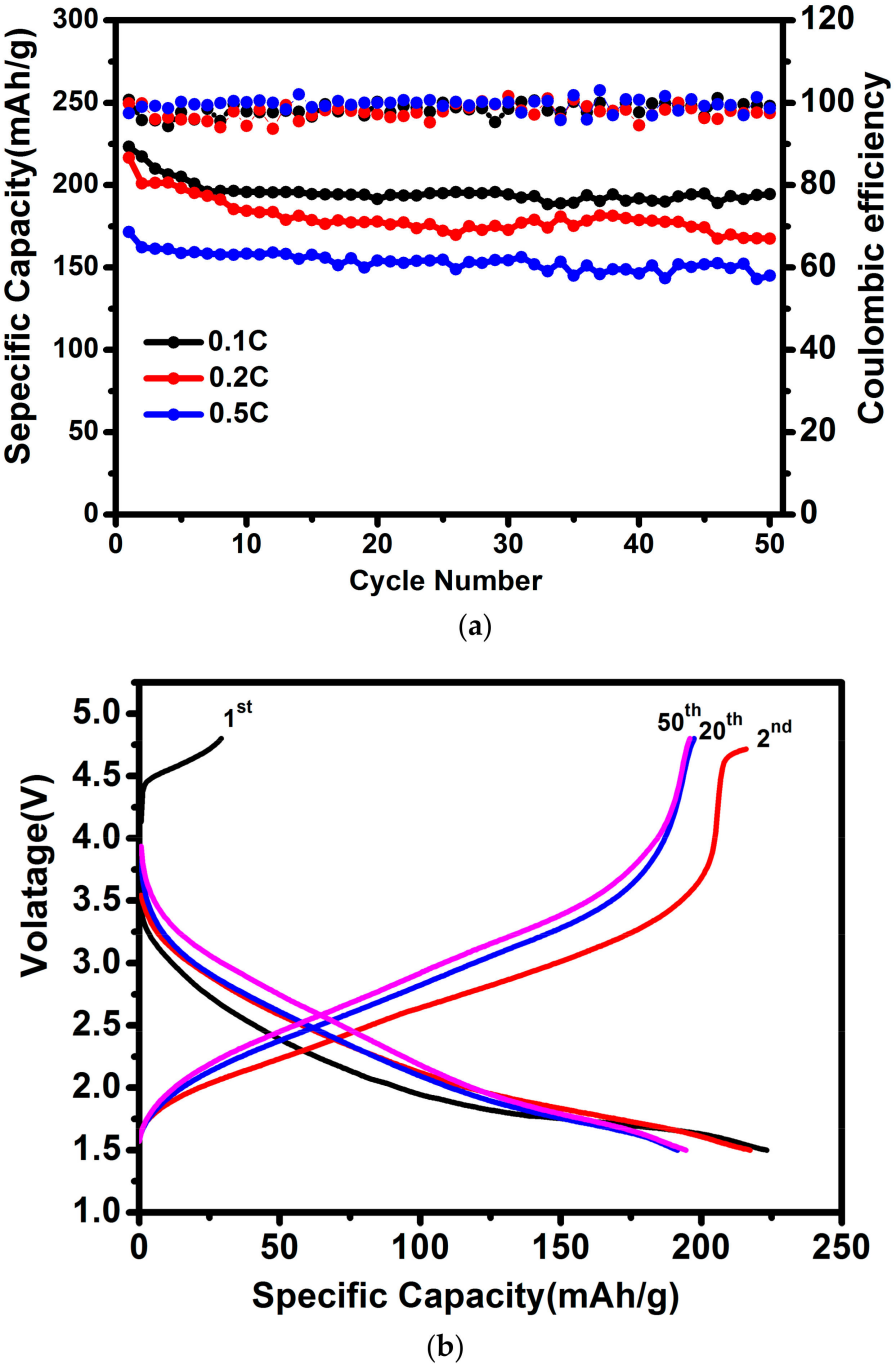
(**a**) Cycling performance; and (**b**) representative charge–discharge curves of the resulting α-LiFeO_2_ samples obtained at 150 °C.

To further evaluate the electrochemical performance, the optimal sample sintered at 150 °C was cycled between 1.5–4.8 V at different rates.Figure 6 shows the cycling performance and corresponding representative charge–discharge profiles of the α-LiFeO_2_ electrodes obtained at 150 °C. It can be seen from Figure 6a that the initial discharge capacity at 0.1 C, 0.2 C, and 0.5 C is 223.3 mAh/g, 216.6 mAh/g and 171.5 mAh/g with capacity retention of 87.1%, 77.3% and 84.5%, respectively. Apart from the first cycle, the Coulombic efficiencies of these α-LiFeO_2_ samples at different rates are very stable and close to 100%, indicating the α-LiFeO_2_ obtained in this work can show excellent reversible electrochemical performance.

Figure 6b displays the representative charge–discharge profiles of this electrode. It can be seen that the charging profile of the first cycle is totally different from the subsequent cycles, and there are disparate profiles of the charge–discharge curves. This is a typical phenomenon for α-LiFeO_2_, which was also found in orthorhombic, corrugated layer, and tetragonal phase LiFeO_2_ [28,29,30]. During the first charging process, the voltage exhibits a sharp increase from the open circuit voltage to 4.25 V. Then, there is a slowly rise to 4.8 V with a long plateau above 4.25 V. When the sample is discharged, the voltage rapidly decreased to 3.0 V and then there is a gradual decrease to the cut-off potential of 1.5 V, giving a long discharge plateau within 1.5–1.8 V. The subsequent discharge curves are consistent with the first cycle, but the subsequent charge curves are totally different from the first one. For the second charging process, the voltage slowly increased from 1.5 V to 3.5 V with a large voltage plateau in the 2.5–3.5 V region, which significantly improves the specific capacity. Then, there is a deep increase with a relative small voltage plateau at 4.25–4.8 V. With continued cycling, this voltage gradually becomes smaller until it disappears after 50 cycles. This typical phenomenon is noted by previous researchers. Morales suggested a structural rearrangement upon lithium removal: The Fe^4+^ atoms, which originally located on octahedral 4a sites might move to tetrahedral 8c position during reaction. This is the main conduction pathway of lithium ions during charging; the displace position of Fe^4+^ may account for the voltage hysteresis and a continuous decrease of plateau at 4.5–4.8 V. Another explanation is that unstable Fe^4+^ may react with the electrolyte during charging process. Wu et al. suggested that the reversible electrochemical reaction occurring in the a-LiFeO_2_ electrode during charge–discharge are Fe^3+^/Fe^2+^, the corresponding reaction formation is:

$${\mathrm{Li}}_{1+x}{\mathrm{FeO}}_{2}\to {\mathrm{LiFeO}}_{2\;}+{\mathrm{xLi}}^{+}+{\mathrm{xe}}^{-}$$

which was also suggested by Kanno et al. and Lee et al. for other LiFeO_2_ polymorphs [4,28].

**Figure 7:**
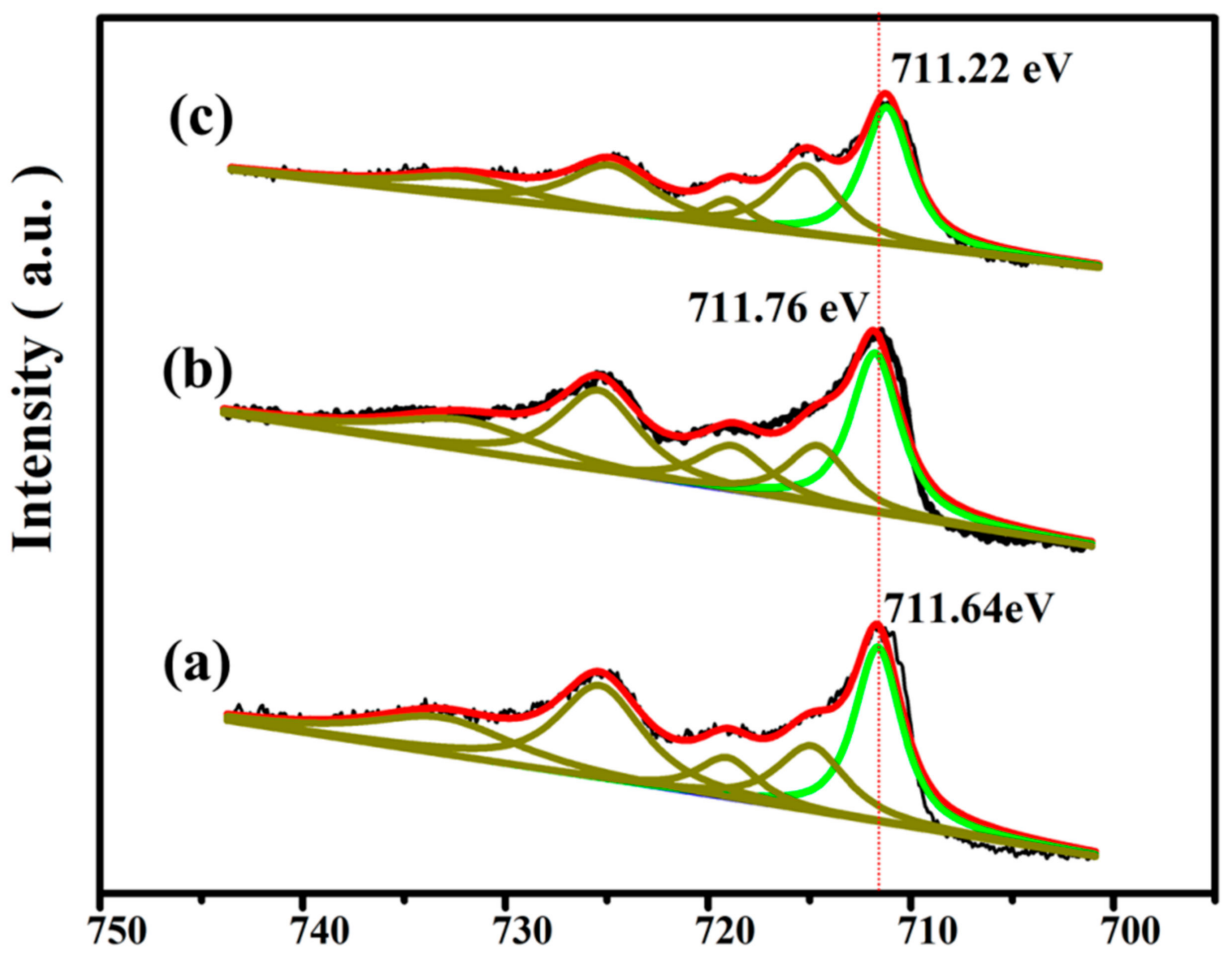
XPS spectra for Fe 2p in α-LiFeO_2_-based electrodes: (**a**) at OCV; (**b**) after one charge process to 4.8 V; and (**c**) after one discharge process to 1.5 V.

The X-ray photoelectron spectroscopy (XPS) was employed to indicate the chemical state of α-LiFeO_2_ at pristine state (OCV), charged to 4.8 V and discharged to 1.5 V. Figure 5 shows the XPS spectra of Fe2p at different states. As shown in Figure 7a–c, the value of binding energy is 711.64 eV, 711.76 eV and 711.22 eV, respectively, which correspond to the binding energy of Fe2p in the research result [31,32]. The binding energy value at OCV is 711.64 eV, which slightly increases to 711.76 eV after charged to 4.8 V, indicating that the environment of Fe is scarcely altered during charging process. The small increase in binding energy can be ascribed to the small fraction of oxidized Fe present [32]. However, the large decrease of binding energy value of 0.42 eV at discharged state (Figure 7c) indicates a prominent change of the Fe oxidation state, which is consistent with the earlier report by Morales et al. [32].

**Figure 8:**
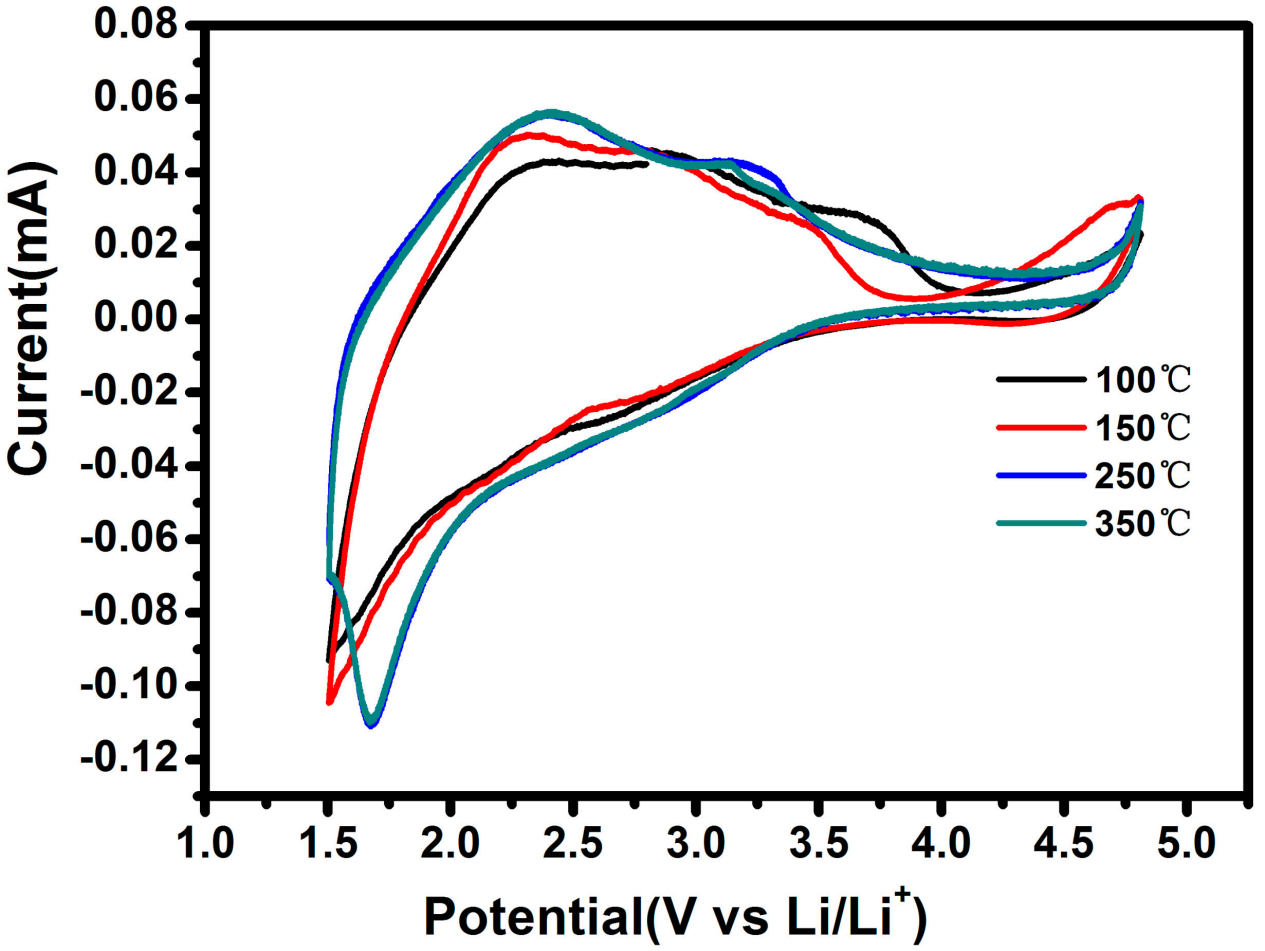
Representative cyclic voltammogram curves of α-LiFeO_2_-based electrodes.

The cyclic voltammetry measurements shown in Figure 8 were used to analyze the oxidation and reduction process during the charge–discharge cycles of all electrodes. The scan rate was 0.1 mV/s, and the potential range is 1.5–4.8 V. The large cathodic peak at 1.75 V corresponds to the voltage plateau of 1.5–1.8 V during the discharge process, in which Li^+^ ions are extraction from α-LiFeO_2_ to form α-Li_1+x_FeO_2_ [33,34]. Two broad anodic peaks at 2.0 and 3.5 V are discovered in all electrodes. They correspond to the voltage plateau in the charge process, in which Li^+^ de-intercalated from α-Li_1+x_FeO_2_ [35]. A sharp rise at 4.8 V is observed in all samples, which further proves that the voltage plateaus above 4.25 V for α-LiFeO_2_. Evidently, among these electrodes, the electrode calcined at 100 °C demonstrates the lowest current densities and smallest peak areas, exposing that the electrode possesses higher inner resistance and poor electrochemical performance [36].

**Figure 9:**
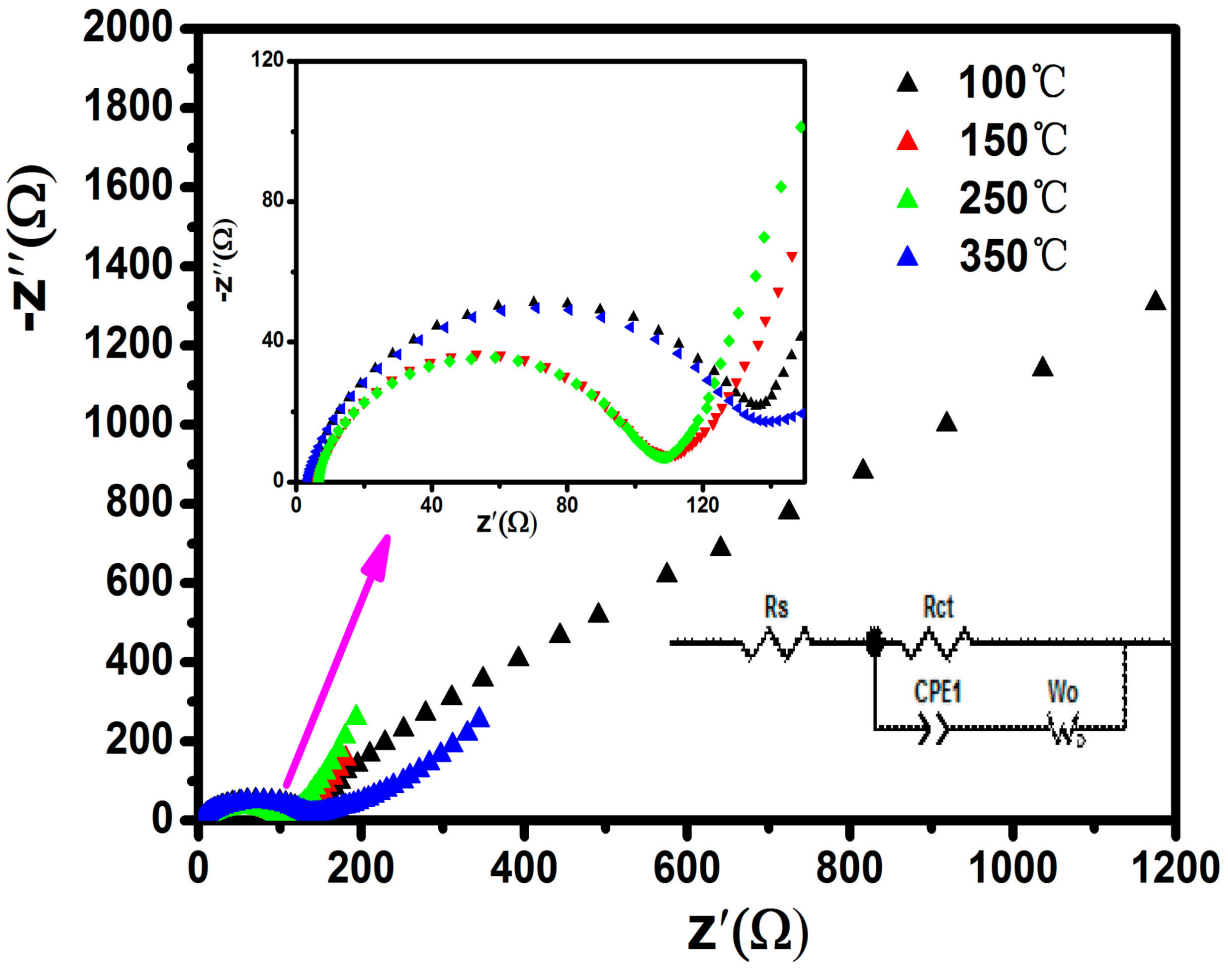
EIS of all electrodes.

Figure 9 presents the EIS of electrodes calcined at different temperature, which displayed one semicircle and a sloping line. The fitting circuit model is displayed in the inset, which includes ohmic resistance (Rs); charge transfer resistance (Rct), which is closely related to the electronic conductivity; double layer capacitance (CPE); and the Warburg impedance (Wo) [37,38]. It can be concluded from Table 1 that the Rct value of electrodes calcined at 100 °C and 350 °C are 152.8 Ω and 121.7 Ω, respectively. The values are higher than electrodes calcined at 150 °C and 250 °C, i.e., 94.4 Ω and 94.59 Ω, respectively. The above results suggest that extremely low and relatively high temperatures would increase the charge transfer resistance.

**Figure 10:**
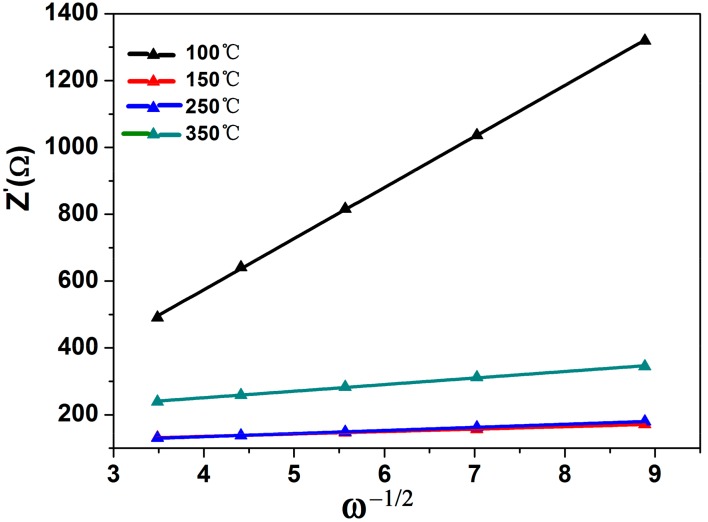
The relationship between $Z^{\prime }$ and ω^−1/2^ in the low frequency range.

The lithium-ion diffusion coefficient can be calculated by the following equation [37].

(1) $$D_{Li^{+}}=\frac{R^{2}T^{2}}{2A^{2}n^{4}F^{4}C^{2}\sigma^{2}}$$

(2) $$Z^{\prime }\propto \sigma \omega^{-1/2}$$

where *R* is the gas constant, *T* is the absolute temperature, *A* is the surface area of the electrode, *n* is the number of electrons per-molecule, *F* is he Faraday constant, *C* is the molar concentration of Li^+^ ions, ω is the angular frequency, and *σ* could been obtained by liner fitting the relationship curve between $Z^{\prime }$ and reprocal square root of the angular frequency in Figure 10 [11,39]. The lithium-ion diffusion coefficients of α-LiFeO_2_ calcined at 100 °C, 150 °C, 250 °C and 350 °C were calculated as 2.98 × 10^−13^, 1.3 × 10^−10^, 8.08 × 10^−11^, and 9.11 × 10^−12^, respectively. The results of calculations are also listed in Table 2. It can be concluded from the electrochemical impedance spectra results that the electrode calcined at 150 °C exhibited smaller Rct and larger D*_Li_*^+^, which may positively affect the electrochemical performance.

## 4. Conclusions

Ultra-small α-LiFeO_2_ nanoparticles with average diameter below 10 nm were successfully prepared via a simple, quick and eco-friendly strategy followed by a low temperature calcine process. The optimal α-LiFeO_2_ calcined at 150 °C for 6 h exhibited excellent electrochemical performance. The initial discharge capacity could reach up to 223.3 mAh/g at 0.1 C, which is close to the theoretical value, and retained 194.5 mAh/g after 50 cycles. It also exhibited high specific capacities of 216.6 mAh/g and 171.5 mAh/g with capacity retention of 77.3% and 84.5% after 50 cycles at 0.2 C and 0.5 C, respectively. Such high electrochemical performance can be attributed to the ultra-small particle size and small charge transfer resistance. The present work may provide a promising strategy to promote the practical application of α-LiFeO_2_.

## Figures and Tables

**Table 1:** The specific surface area and pore volume of samples.

Sample	S_BET_/m^2^·g^−1^	Pore Diameter/nm
100 °C	82.11	8.46
150 °C	194.48	3.27
250 °C	159.30	3.61
350 °C	103.42	8.28

**Table 2:** Fitting values of the electrochemical impedance.

Sample	Rct/Ω	σ/S	D*_Li_*^+^/cm^2^·S^−1^
100 °C	152.8	152.83	2.98 × 10^−13^
150 °C	94.4	7.29	1.3 × 10^−10^
250 °C	94.59	9.28	8.08 × 10^−11^
350 °C	121.7	19.55	9.11 × 10^−12^
